# Kupffer Cells Survive *Plasmodium berghei* Sporozoite Exposure and Respond with a Rapid Cytokine Release

**DOI:** 10.3390/pathogens7040091

**Published:** 2018-11-24

**Authors:** Rebecca E. Tweedell, Le Qi, Zhaoli Sun, Rhoel R. Dinglasan

**Affiliations:** 1 Cellular and Molecular Medicine, Johns Hopkins School of Medicine, Baltimore, MD 21205, USA; rtweedell@gmail.com; 2 W. Harry Feinstone Department of Molecular Microbiology & Immunology, Johns Hopkins Bloomberg School of Public Health, Baltimore, MD 21205, USA; 3 Emerging Pathogens Institute, Department of Infectious Diseases & Immunology, University of Florida, Gainesville, FL 32611, USA; 4 Department of Surgery, Johns Hopkins School of Medicine, Baltimore, MD 21205, USA; lqi7@jhu.edu (L.Q.); zlsun@jhmi.edu (Z.S.)

**Keywords:** Kupffer cell, *Plasmodium berghei*, sporozoite, innate immunity, cell death, malaria, cytokines

## Abstract

The liver stage of the *Plasmodium* life cycle features sporozoite traversal of the liver sinusoidal barrier through Kupffer cells (KCs) followed by invasion of hepatocytes. Little is known about the interaction of *Plasmodium* sporozoites with KCs, the liver-resident macrophages. Previous reports suggest KCs do not mount a pro-inflammatory response and undergo cell death following this interaction. Our work explores this interaction using primary rat KCs (PRKCs) and *Plasmodium berghei* sporozoites. We analyzed PRKC culture supernatants for markers of an immunological response through cytokine arrays. Additionally, cell wounding and death were assessed by monitoring lactate dehydrogenase (LDH) levels in these supernatants and by live/dead cell imaging. We found that PRKCs mount an immunological response to *P. berghei* sporozoites by releasing a diverse set of both pro- and anti-inflammatory cytokines, including IFNγ, IL-12p70, Mip-3α, IL-2, RANTES, IL-1α, IL-4, IL-5, IL-13, EPO, VEGF, IL-7, and IL-17α. We also observed no difference in LDH level or live/dead staining upon sporozoite exposure, suggesting that the KCs are not deeply wounded or dying. Overall, our data suggest that sporozoites may be actively modulating the KC’s reaction to their presence and altering the way the innate immune system is triggered by KCs.

## 1. Introduction

Malaria is a devastating disease that causes over 400,000 deaths per year, mainly among young children in sub-Saharan Africa [1]. *Plasmodium* parasites are responsible for causing disease, and key gaps exist in our understanding of the parasite’s lifecycle. Following transmission from an infected female anopheline mosquito to the skin of a human during blood feeding, the *Plasmodium* parasite makes its way by gliding motility to blood vessels and enters the bloodstream to hone to the first site of invasion and development, the liver [2]. Unlike the cyclical development of *Plasmodium* in erythrocytes, the liver stage (LS) of infection is clinically silent [3]. The LS has not been as well-studied as many other steps in the parasite life cycle. When studying the LS, it is important to consider not only sporozoite invasion of hepatocytes but also the steps leading up to this event. To gain access to hepatocytes, sporozoites must traverse the sinusoidal barrier, which contains liver endothelial cells and Kupffer cells (KCs). It is estimated that at least 60% of *Plasmodium berghei* sporozoites pass through a KC on their way to hepatocytes [4].

KCs, also known as the liver-resident macrophages, make up about 35% of the liver non-parenchymal cells in adult mice [5] and about 30% in humans [6]. They line the liver sinusoids across the Space of Disse from hepatocytes and rapidly clear bacteria and other foreign particles from the blood stream [7]. They also play an important role in promoting immune tolerance in the liver to prevent unnecessary inflammation [8,9,10,11]. However, in cases of high infection levels or liver injury, as demonstrated during *Leishmania* infections, KCs can serve as immune activators [12,13,14]. However, in the case of *Plasmodium* infection, sporozoites can traverse these KCs without being phagocytosed or killed [15]. On the other hand, a recent report highlighted that hepatocyte growth factor (HGF) from KCs of infected mice is essential in promoting apoptosis of *Plasmodium*-infected hepatocytes [16], suggesting that the KCs do produce soluble molecules to affect the overall state of the liver during the infection. Additionally, it was previously shown that an innate immune response can be induced during the LS, contributing to host resistance to reinfection [17], and that leukocytes in the liver can respond to a hepatocyte-propagated type I interferon signal to respond to sporozoite infection [18]. Furthermore, transmission of *Plasmodium* by mosquito bite leads to an increase in the innate immune response when compared to transmission by direct injection of blood stage parasites, suggesting a strong role for the liver’s innate immune system in infection control [19]. However, the full milieu of proteins secreted from KCs upon sporozoite exposure remains unknown. 

When the KC is traversed by the sporozoite, it has been reported that many of the KCs become wounded and succumb to death [4,15,20]. However, signs of collagen secretion and inflammation, which should follow cell wounding and death [21], have not been noted to take place upon sporozoite traversal and infection of the liver. Hepatocytes, which are similarly traversed by sporozoites, are not largely wounded and killed [4,22,23]. These observations imply that the sporozoite is modulating the cellular responses in its favor through a mechanism that is not well understood.

While previous studies have examined downstream effects of sporozoite exposure on the ability of KCs to mount an immune response against a subsequent LPS challenge and have shown down-modulation of the pro-inflammatory response [20], few studies have addressed the KC’s immediate response to sporozoite exposure. Therefore, the true fate and activity of the KC upon traversal remains unclear. Here, we determined the innate immunological response of primary rat KCs (PRKCs) to *P. berghei* sporozoite exposure, and evaluated whether the PRKCs undergo death following exposure. Our work captured a short-lived KC-cytokine secretion profile that was unique to live sporozoite exposure and waned over time while also providing additional evidence that KCs remain viable following exposure to sporozoites.

## 2. Results

### 2.1. PRKCs Secrete a Diverse Array of Cytokines in Response to Sporozoite Exposure

**Figure 1:**
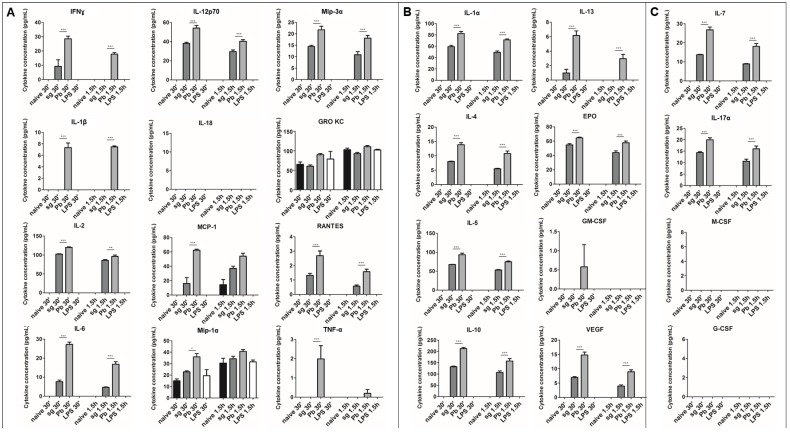
**Cytokine secretion from primary rat Kupffer cells exposed to *P. berghei* sporozoites.** (**A**) M1 cytokines observed in the supernatant of primary rat Kupffer cells isolated using culture method (2) under naïve conditions (naïve), after uninfected salivary gland extract exposure (sg), after *P. berghei* sporozoite exposure (Pb), or after 1 µg/mL LPS treatment (LPS). (**B**) M2 cytokines observed in the supernatant. (**C**) Cytokines associated with both M1 and M2 phenotypes or neither observed in the supernatant. Data represent three biological replicates and two technical replicates with SEM. (Bonferroni’s multiple comparison test, * *p* < 0.05, ** *p* < 0.01, *** *p* < 0.001 comparing sg to Pb).

**Figure 2:**
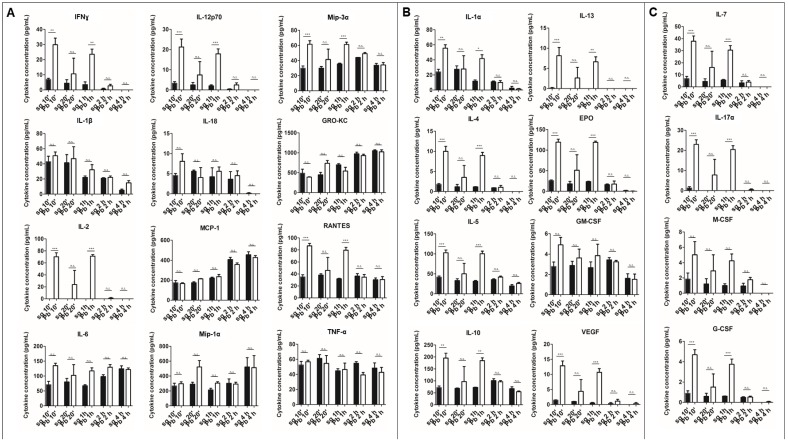
**Cytokine secretion from primary rat Kupffer cells exposed to *P. berghei* sporozoites over time.** (**A**) M1 cytokines observed in the supernatant of primary rat Kupffer cells isolated using culture method (1) at various times after uninfected salivary gland extract exposure (sg) or after *P. berghei* sporozoite exposure (Pb). (**B**) M2 cytokines observed in the supernatant. (**C**) Cytokines associated with both M1 and M2 or neither observed in the supernatant.  Data represent three biological replicates and two technical replicates with SEM. (Bonferroni’s multiple comparison test, * *p* < 0.05, ** *p* < 0.01, *** *p* < 0.001, n.s. not significant).

The cytokine response of KCs to *Plasmodium* sporozoites remains largely unknown. To address this knowledge gap, PRKCs were exposed to *P. berghei* sporozoites, uninfected mosquito salivary gland extracts, or LPS from *Escherichia coli*. We used two different methods for culturing freshly isolated PRKCs in an attempt to minimize the natural death that occurs over time of primary cells kept in culture. Using both culture methods, we observed similar trends (Figure 1 and Figure 2). We observed the secretion of M1 and M2 cytokines (M1: IFNɣ, IL-2, IL-12p70, Mip-3α (CCL20), RANTES (CCL5); M2: IL-1α, IL-4, IL-5, IL-10, IL-13, erythropoietin (EPO), vascular endothelial growth factor (VEGF); both/neither M1/M2: IL-7 (neither), IL-17α (both)) into the PRKC culture supernatants at significantly higher levels following exposure to *P. berghei* sporozoites than the levels observed following exposure to uninfected salivary gland controls. These responses occurred rapidly after the exposure and typically demonstrated a decrease in cytokine level from the 30-min to the 1.5-h time point (Figure 1). Cytokines typically seen after exposure to LPS were not observed with LPS treatment at such early time points. To understand the kinetics of the response more fully and to try our alternative culturing method, we extended our time series to both earlier and later time points. After just 10 min of exposure to sporozoites, PRKCs secreted significantly higher levels of both M1 and M2 cytokines (Figure 2). This increase in cytokine secretion was also seen at 1-h post exposure but waned over the 4-h timeframe.

### 2.2. PRKC Cytokine Secretion is Specific to Live Sporozoite Exposure

**Figure 3:**
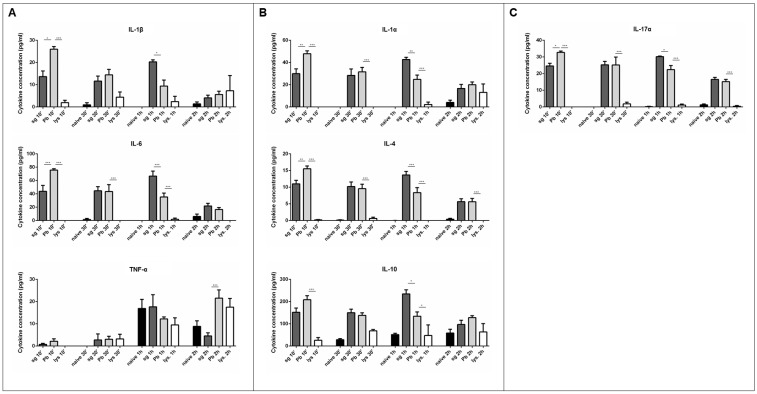
**Cytokine secretion from primary rat Kupffer cells is dependent upon live sporozoite exposure.** (**A**) M1 cytokines observed in the supernatant of primary rat Kupffer cells isolated using culture method (2) after various times under naïve conditions (naïve), after uninfected salivary gland extract exposure (sg), after *P. berghei* sporozoite exposure (Pb), or after lysed *P. berghei* sporozoite exposure (lys.). (**B**) M2 cytokines observed in the supernatant. (**C**) Cytokines associated with both M1 and M2 or neither observed in the supernatant. Data represent three biological replicates and two technical replicates with SEM. (Bonferroni’s multiple comparison test, * *p* < 0.05, * *p* < 0.01, *** *p* < 0.001 comparing sg to Pb and Pb to lys.).

To determine if a live, intact sporozoite is required to activate PRKC cytokine secretion, we compared the cytokine secretion profile resulting from exposure to sporozoite lysate to the cytokine secretion levels observed upon exposure to whole, live sporozoites. We again observed statistically significant increases in levels of cytokine secretion upon exposure to live sporozoites at just 10 min after the exposure, but only noted very low, if any, cytokine secretion after exposure to the lysed sporozoites (Figure 3). The rapid cytokine release waned over time, returning to baseline levels within 2–4 h post exposure. A similar lack of cytokine secretion was observed in response to exposure to paraformaldehyde-fixed (killed) sporozoites (data not shown). To explore the role of parasite traversal in PRKC cytokine secretion, we exposed PRKCs to SPECT2^−^ mutant sporozoites, which are deficient in cell traversal. Exposure to the SPECT2^−^ mutant sporozoites produced slightly lower levels of cytokine secretion than did exposure to wild type sporozoites, though this was not statistically significant in most cases (Supplementary Figure S1).

### 2.3. T cells are not Major Contributors to the Observed Cytokine Secretion

Several cytokines secreted in our assays were unexpected, as they are not stereotypically associated with macrophages (e.g., IL-2). To rule out that these cytokines were emanating from T cell contamination of our PRKCs, we performed FACS analysis following PRKC isolation. We found that our PRKCs were in fact contaminated with approximately 0.4% T cells (Supplementary Figure S2). To understand the effect these T cells may have on our cytokine secretion assay results, we exposed primary rat splenic T cells to *P. berghei* sporozoites. We found that the T cells played no significant role in secreting the cytokines assayed in the PRKCs (Supplementary Figure S3).

### 2.4. PRKCs do not Undergo Increased Levels of Cell Death Following Sporozoite Exposure

**Figure 4:**
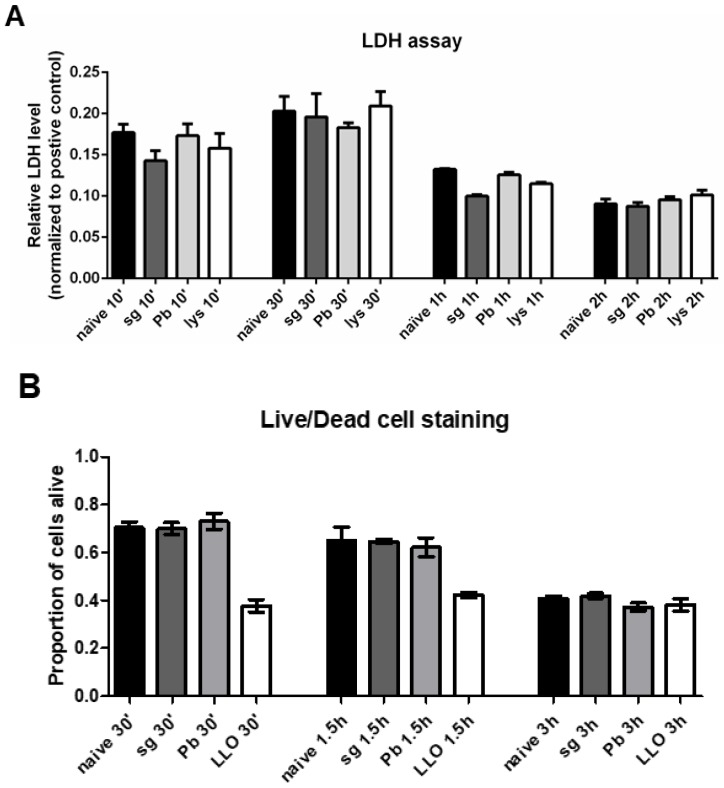
**Primary rat Kupffer cells do not display increased levels of cell death following sporozoite exposure.** (**A**) Lactate dehydrogenase levels in the supernatant of freshly isolated primary rat Kupffer cells isolated using culture method (2) after various times under naïve conditions (naïve), after uninfected salivary gland extract exposure (sg), after *P. berghei* sporozoite exposure (Pb), or after lysed *P. berghei* sporozoite exposure (lys). Data represent three biological replicates with two technical duplicates with SEM. All data is normalized to the positive control at 1.0. (**B**) Live/dead cell imaging was performed on thawed, cryopreserved primary rat Kupffer cells after various times under naïve, baseline conditions (naïve), after uninfected salivary gland extract exposure (sg), after *P. berghei* sporozoite exposure (Pb), or after listerolysin O exposure (LLO); the number of live and dead cells were counted. Data represent three biological replicates with five microscope fields counted for each.

Given that previous reports have suggested that KCs undergo apoptosis and cell death following sporozoite exposure [20], we measured the levels of lactate dehydrogenase (LDH) in the supernatants of freshly isolated PRKCs exposed to stimuli. We did not see an increase in the level of LDH following exposure to *P. berghei* sporozoites (Figure 4A), suggesting that the cells were not being significantly wounded or killed. We also performed an alternative live/dead cell staining approach using thawed, cryopreserved PRKCs (cell purity ≥ 90% before cryopreservation) and obtained similar results, with no significant difference observed between the proportion of live cells present under naïve conditions vs exposure to *P. berghei* sporozoites (Figure 4B, Supplementary Table S1).

## 3. Discussion

Our data demonstrate that PRKCs can mount a rapid and diverse cytokine response to sporozoite exposure. This PRKC response is remarkable since exposure to the well-characterized KC activator LPS resulted in more protracted release kinetics; TNF-α secretion from PRKCs, for example, does not increase until the 4–12-h timeframe (Figure 1, Supplementary Figure S4). However, our findings are consistent with the observations that traversal of cells by sporozoites occurs on the scale of minutes [4] and that just 5 min is long enough for macrophages to begin an immune response [24,25]. Additionally, the response to sporozoite exposure was markedly different from the response to uninfected salivary gland extract. While low levels of pro- and anti-inflammatory cytokines were secreted in response to the uninfected salivary gland extract, secretion was statistically significantly higher upon exposure to sporozoites at early timepoints for the M1-associated cytokines IFNγ, IL-12p70, Mip-3α, IL-2, and RANTES; the M2-associated cytokines IL-1α, IL-4, IL-5, IL-13, EPO, and VEGF; and the non-M1-M2 partitioning cytokines IL-7 and IL-17α (Figure 1 and Figure 2). Some additional cytokines, such as IL-1β, displayed significantly altered secretion levels under only one of the two culture conditions used, preventing conclusive findings for these cytokines. 

One important limitation of our study is the variation in the absolute value of the cytokine levels from experiment to experiment. These variations are likely due to culture-specific phenotypes, differences between the donor rats from which the KCs were isolated, and/or sporozoite and mosquito lot to lot variation. It is also important to remember that despite the use of rigorous dissection methods similar to those used in other immune assays like the ILSDA [26], matched levels of mosquito contaminants will always be present in the sporozoite and uninfected salivary gland pools [27,28], which can cause variability in the background KC cytokine secretion from experiment to experiment. Additionally, the in vivo implications of the specific quantities of cytokines produced remain unclear. In spite of these limitations, statistically significant differences between secretion levels from sporozoite-exposed and uninfected salivary gland-exposed PRKCs are robustly replicated between experiments for IFNγ, IL-12p70, Mip-3α, IL-2, RANTES, IL-1α, IL-4, IL-5, IL-13, EPO, VEGF, IL-7, and IL-17α.

The rapid cytokine secretion response is also short-lived, with cytokine levels dropping to control levels within 2–4 h. It has long been known that IL-1β and IL-18 can be stored in the cell as a soluble, inactive form that is activated for rapid secretion and response [29], but all other cytokines studied herein do not share this phenotype. This may suggest that the KCs do in fact have additional stored cytokine stocks that can be released immediately, as opposed to what has been observed for a targeted, pathogen-associated molecular pattern (PAMP)-activated response. Alternatively, the parasite may be interfering with calcium signaling within the cells, causing a rapid cytokine release that is not maintained as calcium signaling returns to normal over a 2–4-h timeframe following traversal [30,31]. If a sustained production of cytokines were occurring, we would expect to see an increase in secretion again by the later time points; however, this was not observed (Figure 1 and Figure 2). Additionally, the secreted cytokines come from both MyD88-dependent and MyD88-independent pathways [32,33,34,35]. These data, along with the observation of both M1 and M2 cytokine secretion in our assays, suggest to us that the cytokine response may be non-specific and not reliant on any particular TLR signaling path; further testing of this hypothesis will be needed. Previous studies in *Plasmodium* have shown that glycosylphosphatidylinositol anchors can stimulate TLRs and that innate immune cells can recognize TLR ligands [36], though the exact roles of these processes during the pre-erythrocytic stages have not been well characterized.

Although many of the cytokines and chemokines that we have measured in our assays are not frequently associated with KC responses, they have been shown to be secreted by KCs and/or macrophages in previous studies, i.e. RANTES, EPO, IL-5, VEGF, and M-CSF [33,37,38,39,40,41]. Of particular note, IL-7 is a cytokine that has not been previously associated with secretion by KCs. IL-7 is known to be associated with secretion from hepatocytes [42], but our KC isolation method excludes the collection of hepatocytes, and hepatocytes were never observed by microscopy in the KC cultures used. We interpret the data as suggesting that KCs may also contribute to IL-7 levels in the liver, though it is important to note that this observation requires further study. Additionally, we cannot completely rule out the possibility that a small number of capsular macrophages, a relatively recently recognized but poorly-described cell population [43], or perivascular macrophages could have been present in our KC preparations.

Only live, whole sporozoites triggered this increased secretion of the diverse cytokine profile. Cytokine secretion in response to lysed sporozoites was even lower than secretion in response to uninfected salivary gland extracts, likely because any other immune stimuli present underwent the same lysis procedure as the sporozoites, altering their stimulatory capabilities. Similarly, exposure to the traversal-deficient SPECT2^−^ mutant sporozoites produced slightly lower cytokine secretion than did wild type sporozoites; however, this trend did not achieve significance for most cytokines (Supplementary Figure S1), leaving open the possibility that traversal of KCs is not required for their innate immune activity. Overall, these data highlight the importance of parasite factors that can actively influence the PRKC innate immune response. For example, it has been previously shown that circumsporozoite protein binds the low-density lipoprotein receptor-like protein 1 on KCs to induce a cAMP-dependent signaling pathway to suppress the oxidative burst normally used to kill pathogens [44]. It has also been shown that the *Plasmodium* sporozoite protein essential for cell traversal (SPECT) putatively interacts with KC surface proteins to facilitate cell traversal [45]. It is likely that many other poorly characterized *Plasmodium* proteins have roles that strongly affect KC function and activation.

Finally, in contrast to previous reports [20], our data suggest that KCs are not wounded significantly or killed by sporozoite exposure (Figure 4, Supplementary Table S1). It is important to note that the live-dead imaging was carried out with cryopreserved PRKCs (Figure 4B, Supplementary Table S1), which have been previously noted to have ~60% recovery after thawing [46], affecting the baseline levels of cell death observed. Two major differences between previous work on KC wounding and death in response to sporozoites and our study are the species and number of sporozoites used to interact with the KCs. In our study, we used *P. berghei* at a ratio of 1 sporozoite to 1 KC, while previous work on KC response to sporozoites used *P. yoelii* at a ratio of 1 sporozoite to 1 KC and 3 sporozoites to 1 KC [20]. Memory CD8 T cell effector paths for targeting *P. berghei* and *P. yoelii* sporozoites are quite different [47], so it is likely there would also be species to species variation in how KCs respond to *Plasmodium* sporozoites. Additionally, the higher numbers of sporozoites interacting with and traversing each KC may have caused more damage, resulting in cell death; in fact, previous work has shown that the use of a 1:1 ratio results in notably lower rates of cell death compared to the 3:1 ratio [20].

Overall, our study suggests that the KC–sporozoite interaction is not silent. KCs mount a rapid, short-lived cytokine secretion response without being largely wounded or killed. This work opens the door to answering further questions about this essential step in the parasite’s life cycle. Finding ways to tailor the KC’s response towards a more effective activation of the body’s immune system could potentially eliminate the parasite’s ability to enter the liver so discreetly and aid in malaria eradication efforts.

## 4. Materials and Methods

### 4.1. Cells

All animals and experimental protocols used in this study were approved by the Johns Hopkins Animal Care and Use committee and the IACUC, and the methods were carried out in accordance with IACUC and institutional guidelines and regulations. PRKCs were obtained commercially (cryopreserved rat KCs, ThermoFisher Scientific, Waltham, MA, USA) for live-dead cell imaging assays or were freshly isolated from rats using a protocol adapted from Dr. Zhaoli Sun [48] for cytokine secretion and LDH assays. It has been previously demonstrated that cryopreserved KCs maintain their phenotypic features and can be successfully used as surrogates for freshly isolated cells [46]. 

### 4.2. Primary Cell Isolation and Purification

To obtain cells for cytokine secretion and LDH assays, male Lewis rats (150–250 g) were used. Rat KCs were chosen over those of mice due to the large number that can be obtained per animal. Rats were anesthetized with isoflurane; the abdomen was dissected, and the portal vein was cannulated. The liver was then perfused with 100 mL Hank’s Buffered Saline Solution (HBSS) for 5 min. The inferior vena cava was cut to allow the fluid to drain. The liver was then perfused with 100 mL 0.05% collagenase (Type IV from *Clostridium histolyticum*; Sigma-Aldrich, St. Louis, MO, USA) solution in HBSS over 7–10 min. The liver was then removed from the body cavity and washed with HBSS + 30 mM HEPES + 0.1% calcium chloride. The tissue was mashed in HBSS + 30 mM HEPES + 0.1% calcium chloride with 0.05% collagenase, 0.1 mg/mL DNase, and 0.2 mg/mL Pronase. Connective tissue was removed, and the liver cell suspension was incubated at 37 °C for 15 min with agitation. Cells were then spun at 300× *g* for 5 min 3 times and washed with HBSS + 30 mM HEPES + 0.1% calcium chloride + 0.05 mg/mL DNase + 1000 units/mL penicillin + 1000 µg/mL streptomycin after each spin. Cells were then spun at 100× *g* for 1 min to pellet hepatocytes; the pellet was discarded. The supernatant was collected and spun at 300× *g* for 5 min. The cell pellets were resuspended in HBSS + 30 mM HEPES + 0.1% calcium chloride and applied to a Percoll gradient (15 mL 50% Percoll solution, 15 mL 25% Percoll solution, 15 mL cell suspension). The gradients were spun at 800× *g* for 15 min. The top fraction of the spun gradient was removed, and PRKCs were collected from the second fraction and washed twice with HBSS + 30 mM HEPES + 0.1% calcium chloride. The final cell pellets were then resuspended in complete RPMI media (cRPMI), defined as RPMI + 10% heat-inactivated fetal bovine serum (HIFBS) + 1 × MEM amino acids solution (ThermoFisher Scientific, Waltham, MA, USA) + 1000 units/mL penicillin + 1000 µg/mL streptomycin, and used for plating following two different methods. (1) Cells were plated on T75 flasks that had been coated for 48 h with HIFBS. After cells had adhered in a humidified chamber at 5% CO_2_ and 37 °C for an hour, the flasks were washed with HBSS + 30 mM HEPES + 0.1% calcium chloride 3 times, and adherent cells were lifted from the flasks in cold PBS on ice for 1–2 h. This cell suspension was then used to plate cells in cRPMI for 12–16 h before assays. (2) Cells were plated directly for assays on 24- or 48-well plates previously coated for 48 h with HIFBS; cells were allowed to adhere for 2 h in a humidified chamber at 5% CO_2_ and 37 °C and washed 3 times with HBSS + 30 mM HEPES + 0.1% calcium chloride before assays. All assays were performed in cRPMI.

Primary rat T cells were obtained from the matched PRKC donor rat. Rat spleens were homogenized in cRPMI. Cells were then passed through a 21-guage needle in a 5 mL syringe to filter out tissue clumps. Cells were spun at 300 × g, 7 min. The cell pellet was resuspended in red blood cell lysis buffer (ammonium chloride buffer) and incubated at room temperature for 10 min with agitation. Cells were spun at 300 × g, 5 min, and the cell pellet was resuspended in cRPMI. This cell suspension was then allowed to adhere to plates to remove adherent cells. The supernatant was collected, and cells were pelleted and resuspended in MACs buffer (Miltenyi Biotec). Pan-T cell MicroBeads (Miltenyi Biotec) were added to the cell suspension and incubated on ice for 15 min. The cells were then suspended in 500 µL MACs buffer and run through an LS column on a MACs magnet (Miltenyi Biotec). T cells were collected from the elution of cells that bound to the column in the magnet. 

### 4.3. FACS Analysis for T Cell Quantification

PRKCs and primary rat T cells were used fresh after isolation; PRKCs were isolated and enriched using method (1) above with cells being taken for FACS analysis after lifting in PBS on ice and before the final plating step. PRKCs and T cells were blocked with anti-FcɣR II/III to prevent nonspecific binding. Cells were then stained with anti-F4/80-PE-Cy7 and anti-CD3-AF488. Propidium iodide was used to determine cell viability. Cells were analyzed on a DakoCytomation MoFlo (Beckman Coulter) with the following detection filters: FL1/AF488 (530/30), FL2 (580/30), and FL3/PE-Cy7 (740 L). Wavelengths are reported in nm. Gating was performed to exclude cellular debris by using the forward scatter and side scatter gates. From the population of cells, viable cells were selected by gating for propidium iodide negative cells. Due to the high autofluorescence of KCs, a gating strategy using FL1/AF488 vs FL2, instead of FL3/PE-Cy7, was used for subsequent gating to identify the T cell population. For more detail on the gating strategy, see Supplementary Figure S2.

### 4.4. Sporozoite Generation and Collection and Uninfected Salivary Gland Extract Collection

*Anopheles stephensi* (day 6–10) mosquitoes were fed on a mouse infected with *P. berghei* mCherry parasites or *P. berghei* SPECT2^−^ parasites (a mutant deficient in cell traversal) exhibiting 0.5–2 exflagellations per field under a 40× objective. Fully fed mosquitoes were dissected 18–24 days post-feed to collect salivary gland sporozoites, based on the standard method for inhibition of liver stage development assays [26]. Unfed mosquitoes reared in parallel were dissected simultaneously to collect uninfected salivary gland extracts to use as a control to account for mosquito proteins and other potential mosquito-derived contaminants that cannot be separated from sporozoites despite the most rigorous washing and purification steps [27,28]. Mosquitoes were collected in 70% ethanol, then transferred to 1 × PBS in a petri dish on ice; salivary gland pairs were dissected and collected into 500 µL cRPMI on ice; the glands were lightly spun for 3 min at 1200× *g* and then crushed by hand with a plastic, sterile pestle; this 500 µL crushed preparation was then filtered through glass wool before use. Both sporozoites and infected salivary gland extracts were then diluted identically at least 10-fold in sterile cRPMI before being added to cells in a volume of 500 µL in 24-well plates or 200 µL in 48-well plates. Sporozoites were used at a ratio of 1 sporozoite:1 PRKC, and the analogous volume of uninfected salivary gland extract was used. Although we noted that direct salivary gland dissections (as opposed to commonly used thorax-dissections) produced sporozoites without any large mosquito cellular debris, these sequential “dilution-washing” steps were included to “dilute” the potential gland-derived contaminants from the sporozoites; especially since low rcf centrifugation does not effectively pellet sporozoites that have been released from salivary glands, and higher rcf negatively impacts sporozoite viability/activity. Lysis of sporozoites was achieved by rapid freezing and thawing of sporozoites in liquid nitrogen (1 min) and a 37 °C water bath (4 min) 5 times. Fixation-mediated killing of sporozoites was achieved by incubating the released sporozoites in 4% paraformaldehyde for 20 min at room temperature. The sporozoites were then further washed in 1 mL cRPMI and spun to remove excess paraformaldehyde that could otherwise affect the KC response.

### 4.5. Bio-Plex Cytokine Assays

Cells were plated in 24- or 48-well plates following methods (1) or (2) outlined in the “Primary Cell Isolation and Purification” section. The same size plate was used for all samples within a given experiment, and in experiments where secretion from T cells and PRKCs are compared, the same number of cells were plated for each cell type. Cells were exposed to no stimuli (naïve), uninfected mosquito salivary gland extracts (sg), *P. berghei* mCherry sporozoites at a ratio of 1 sporozoite to 1 cell (Pb), *P. berghei* SPECT2^−^ sporozoites at a ratio of 1 sporozoite to 1 cell (SPECT2^−^), 1 µg/mL LPS from *E. coli* (LPS), lysed *P. berghei* mCherry sporozoites at a ratio of 1 sporozoite to 1 cell (lys.), or *P. berghei* mCherry paraformaldehyde-fixed sporozoites at a ratio of 1 sporozoite to 1 cell (fPb) and incubated in a humidified chamber at 5% CO_2_ and 37 °C. The ratio of 1 sporozoite to 1 cell was chosen to most closely mimic the natural in vivo conditions of the KC-sporozoite interaction. After the appropriate amount of time, the supernatant from the culture was removed and spun at 12,000× *g* for 10 min to remove any cellular debris before being used for analysis. Supernatants were analyzed using the Bio-Plex cytokine array platform following manufacturer’s instructions (Bio-Rad). Briefly, the cytokine assay standard(s) were reconstituted on ice for 30 min. A fourfold dilution series of the standard was made. The Bio-Plex magnetic beads were added to the assay plate and washed twice. Standards, blanks, and supernatants were then added to the plate and allowed to bind the beads for 1 h. The plate was washed three times, and then detection antibodies were added for 30 min. The plate was washed three times, and then Streptavidin-PE was added for 10 min. The plate was washed three times again, and then the beads were resuspended in assay buffer to be read on a Bio-Plex 200 instrument (Bio-Rad) using high PMT or the Luminex MAGPIX instrument. Standard curves for each cytokine were analyzed and optimized in the Bio-Plex Manager software. PRKC supernatants were run in biological triplicate with technical duplicates. T cell supernatants were run with technical duplicates.

### 4.6. Lactate Dehydrogenase (LDH) Assay

The lactate dehydrogenase assay (Pierce LDH cytotoxicity assay kit) was performed following the manufacturer’s protocol using freshly isolated PRKCs. We determined in our initial pilot experiments that the dextran assay, commonly used to determine hepatocyte cell fate following sporozoite cell traversal [49], is inappropriate for our system. We observed that naïve PRKCs take up dextran without any stimulus, which would significantly compromise qualitative/quantitative measures of cell viability. We collected supernatants from PRKCs exposed to no stimuli (naïve), uninfected salivary gland extracts (sg), *P. berghei* sporozoites (Pb), or lysed *P. berghei* sporozoites (lys.) for use in the LDH assay. The reaction mix from the kit was added to the supernatants and incubated for 30 min. Stop solution was then added, and the absorbance at 490 nm and 680 nm were measured. The background 680 nm absorbance was subtracted from each read, and the output was normalized to the kit positive control (cells + lysis buffer) at 1.0.

### 4.7. Live/Dead Cell Imaging Assay

Due to the existence of multiple forms of cell death that can involve many different pathways [50], we included an orthogonal assay to measure KC viability following sporozoite exposure. The LDH assay typically detects cells undergoing necrosis, but not necessarily other forms of cell death such as apoptotic, quasi-apoptotic, and nonapoptotic mechanisms [51,52]. Additionally, the LDH assay focuses on a population of cells instead of single-cell death events, which can provide additional information about the cell death process [53]. Therefore, to supplement the LDH assay, we included an additional live/dead assay that allows single-cell analysis and is not necrosis-specific through the use of a cell permeable calcein AM that measures both intracellular esterase activity and membrane integrity. When a cell is alive and has an intact membrane, calcein AM enters the cell and is converted to fluorescent calcein by intracellular esterase activity, while a dead cell with a damaged membrane takes up the cell impermeable nuclear dye. The ThermoFisher Live/Dead cell imaging kit was used following the manufacturer’s protocol. Commercially purchased cryopreserved rat KCs (ThermoFisher Scientific, Waltham, MA, USA; cell purity ≥ 90%) were thawed and plated directly into 24-well plates on collagen-coated coverslips overnight before beginning the assay. KCs were then exposed to no stimuli (naïve), uninfected salivary gland extracts (sg), *P. berghei* mCherry sporozoites (Pb), or 387.5 ng/mL listeriolysin O, a pore forming toxin (LLO). LLO was used as a positive control to confirm that the assay could appropriately identify dead cells and differentiate live cells that had taken up the cell impermeable dye in a phagosome from dead cells that had the dye in the nucleus. After 15 min, the live/dead imaging reagent mix was added to the cells. Cells were imaged, and the number of live (fluorescing green) and dead (fluorescing red in the nucleus) cells was counted at 30 min, 1.5 h, and 3 h following cell exposure to stimuli. Cells with red fluorescence in phagosomes but not in the nucleus were not counted as dead cells. Morphology to examine cell size and shape, key distinguishing features between PRKCs and other commonly contaminating liver cells such as hepatocytes and stellate cells, was used to confirm cell purity. For each stimulus, three biological replicates were analyzed, and five microscope fields of each replicate were counted at each time point using the 10× objective on the EVOS Cell Imaging System (ThermoFisher Scientific, Waltham, MA, USA).

## Supplementary Materials

Supplementary Materials are available online at http://www.mdpi.com/2076-0817/7/4/91/s1. These include Supplemental Figure S1, Cytokine secretion from primary rat Kupffer cells exposed to traversal-deficient SPECT2^−^ sporozoites; Supplemental Figure S2, FACs analysis of isolated rat liver cells to determine the percentage of T cells in the population; Supplemental Figure S3, Cytokine secretion from primary rat Kupffer cells and T cells exposed to *P. berghei* sporozoites; Supplemental Figure S4, Cytokine secretion from primary rat Kupffer cells in response to LPS and IFNγ; and Supplemental Table S1, Primary rat Kupffer cell death following exposure to stimuli.
