# Supplementary material for: Kupffer Cells Survive Plasmodium berghei Sporozoite Exposure and Respond with a Rapid Cytokine Release
Source: Pathogens. 2018 Nov 24;7(4):91. doi: 10.3390/pathogens7040091 (PMC6313776; doi:10.3390/pathogens7040091)
Supplement: Supplementary file 1 [file pathogens-07-00091-s001.pdf]

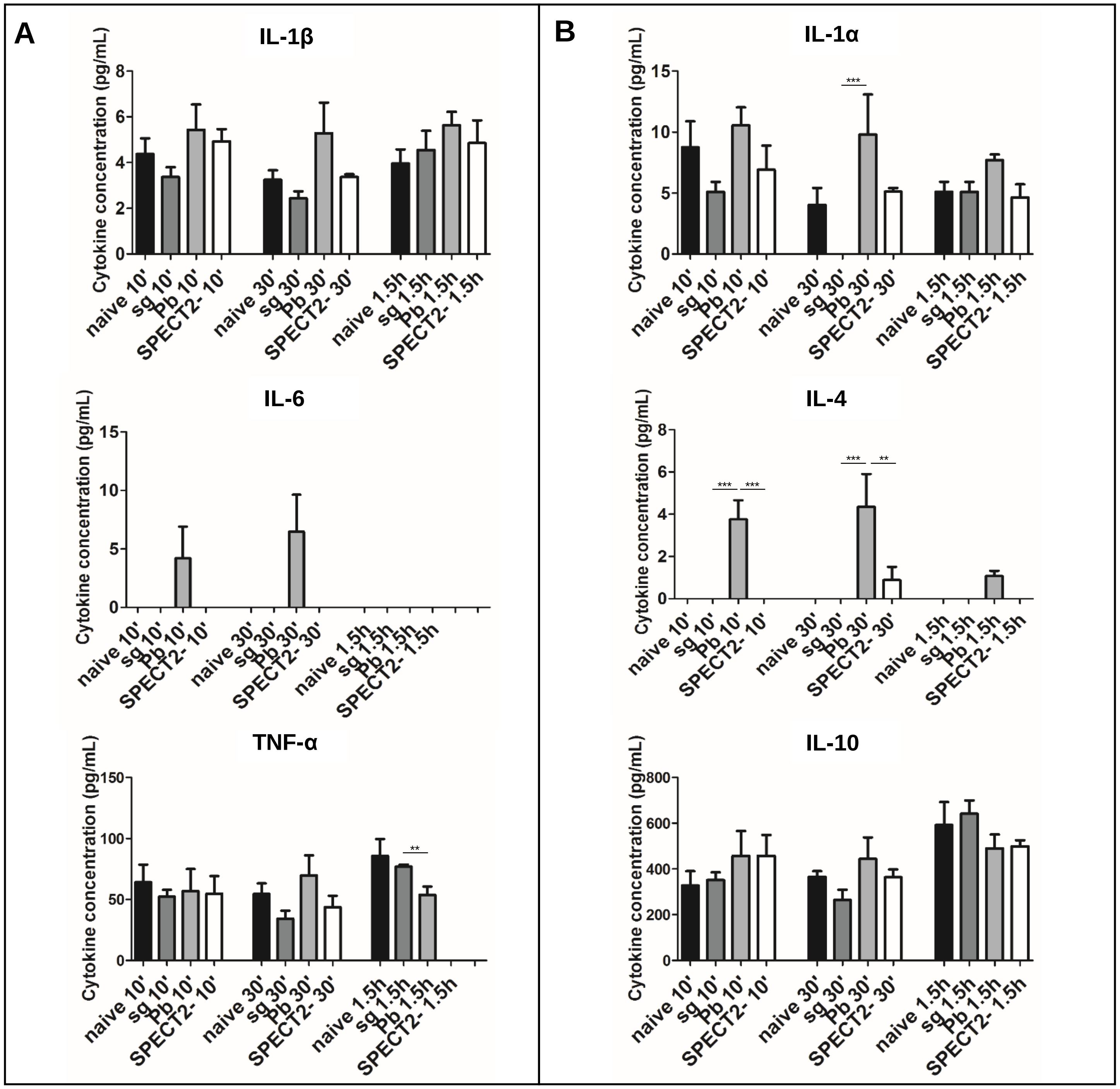

Figure S1. Cytokine secretion from primary rat Kupffer cells exposed to traversal-deficient SPECT2$^-$ sporozoites. A) M1 cytokines observed in the supernatant of primary rat Kupffer cells isolated using culture method (2) after various times under naïve conditions (naïve), after uninfected salivary gland extract exposure (sg), after *P. berghei* sporozoite exposure (Pb), or after SPECT2$^-$ *P. berghei* sporozoite exposure (SPECT2-). B) M2 cytokines observed in the supernatant. Data represent three biological replicates and two technical replicates with SEM. (Bonferroni's multiple comparison test, **p*<0.05, **p*<0.01, ****p*<0.001 comparing sg to Pb and Pb to SPECT2$^-$)

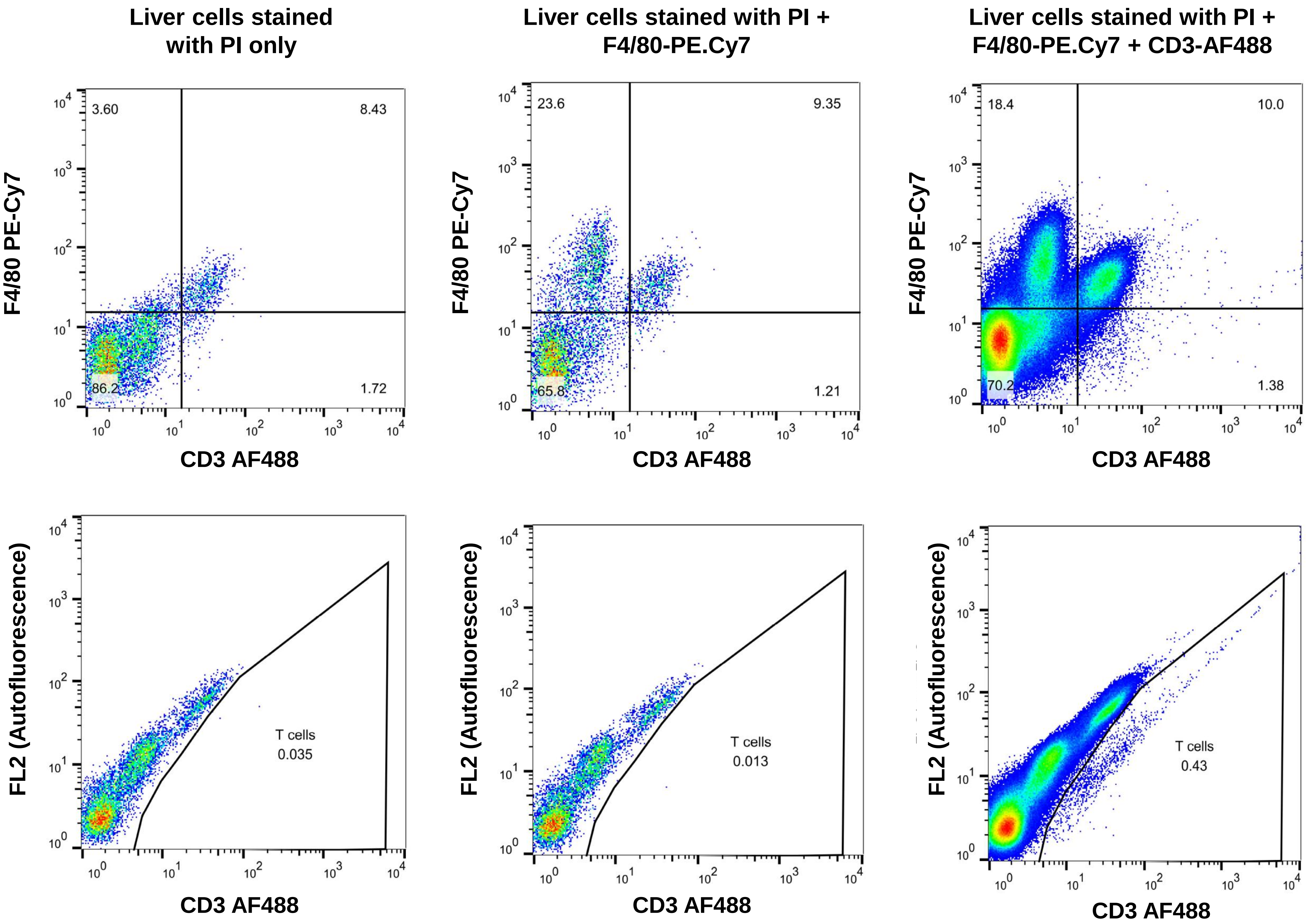

Population	Count	%
Total	8580	100
T cells	3	0.035

Population	Count	%
Total	7793	100
T cells	1	0.013

Population	Count	%
Total	248063	100
T cells	1072	0.43

Figure S2. FACS analysis of isolated rat liver cells to determine the percentage of T cells in the population. Liver cells isolated from rats were FACS sorted using the F4/80-PE.Cy7 dye to stain for Kupffer cells and the CD3-AF488 dye to stain for T cells. Because Kupffer cells display high levels of autofluorescence, as seen by the presence of $AF488^{+}PE.Cy7^{+}$ cells in the unstained liver cell population (top left panel), a gating strategy that accounted for this autofluorescence was needed to most accurately define the T cell population. Unstained cells were run and gated using FL1 (AF488) vs FL2 to identify where the autofluorescence falls of the scatter plot; based on this gating strategy, the unstained cells form a diagonal line (bottom left panel). Cells that fall to the right of that diagonal line are the true $CD3\text{-}AF488^{+}$ cells. Therefore, the gated region on the lower panels denotes where T cells fall on the scatter plot. Since there is some overlap in the FL1 and FL2 filters, this $CD3\text{-}AF488^{+}$ cell population also forms a diagonal distribution (bottom right panel; higher FL2 expression accompanies higher AF488 expression). Approximately 0.4% of cells in the primary rat Kupffer cell isolation are contaminating T cells. Left and center panels are representative images from the validation of the gating strategy on the control cell populations; the right panels are representative images from the test cell population.

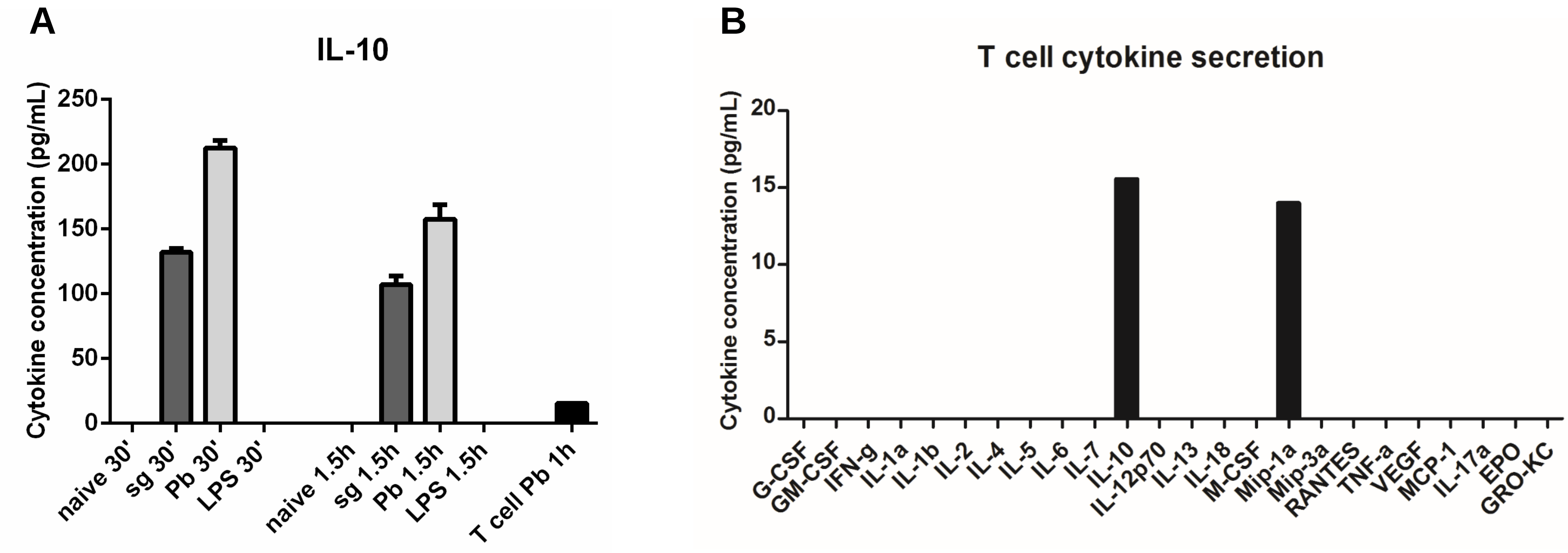

Figure S3. Cytokine secretion from primary rat Kupffer cells and T cells exposed to *P. berghei* sporozoites. A) IL-10 cytokine levels observed in the supernatant of 50,000 primary rat Kupffer cells or 50,000 purified primary rat T cells after various times under naïve conditions (naïve), after *P. berghei* sporozoite exposure (Pb), after uninfected salivary gland extract exposure (sg), or after LPS exposure (LPS). Kupffer cell data represent three biological replicates with SEM. T cell data represents one biological replicate and two technical replicates. B) Levels of various cytokines observed in the supernatant of 50,000 primary rat T cells after 1 hour of *P. berghei* sporozoite exposure.

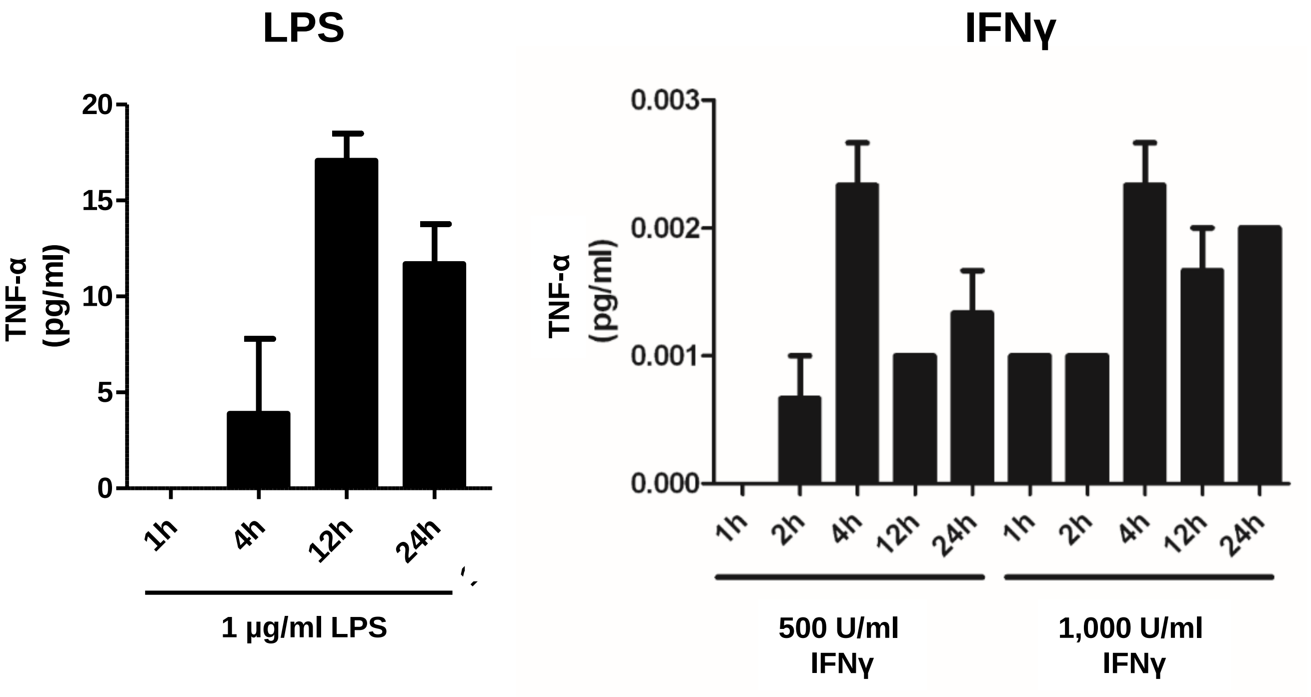

Figure S4. Cytokine secretion from primary rat Kupffer cells in response to LPS and IFNγ. Primary rat Kupffer cells were exposed to 1 µg/ml LPS, 500 U/ml IFN-γ, or 1000 U/ml IFNγ. Supernatants were analyzed for the amount of TNF-α secreted at various time points after exposure. Data represents 3 biological replicates with SEM.

Treatment	naïve 30'		sg 30'		Pb 30'		LLO 30'		naïve 1.5h		sg 1.5h		Pb 1.5h		LLO 1..5h		naïve 3h		sg 3h		Pb 3h		LLO 3h	
	green (live)	red (dead)	green (live)	red (dead)	green (live)	red (dead)	green (live)	red (dead)	green (live)	red (dead)	green (live)	red (dead)	green (live)	red (dead)	green (live)	red (dead)	green (live)	red (dead)	green (live)	red (dead)	green (live)	red (dead)	green (live)	red (dead)
Coverslip 1	41	9	19	9	17	8	10	23	14	13	36	14	70	8	41	53	10	16	17	33	17	15	17	27
	8	3	20	10	16	10	13	29	8	10	41	16	18	8	45	56	11	16	11	15	16	38	4	12
	11	5	19	10	38	10	15	20	13	11	26	19	44	19	44	64	6	12	8	14	19	26	5	15
	16	6	33	15	33	19	8	25	10	10	26	21	28	28	53	53	10	15	15	30	17	36	13	22
	11	6	15	12	17	9	5	9	13	5	24	9	17	13	12	17	19	22	35	35	42	64	22	27
Coverslip 2	17	6	31	12	14	7	31	39	20	12	23	5	17	14	24	37	19	27	20	25	16	41	45	50
	14	4	22	9	15	4	19	32	15	7	16	15	21	21	35	42	24	22	21	33	12	20	26	38
	18	10	33	13	31	12	7	10	14	9	13	6	23	19	26	29	11	25	10	13	15	37	22	24
	37	17	16	10	69	6	4	8	43	12	32	19	34	24	12	23	28	30	12	15	16	27	13	23
	33	10	34	13	25	12	11	16	35	9	16	14	36	9	9	18	11	21	12	20	27	42	12	20
Coverslip 3	44	30	50	15	30	7	25	46	28	14	19	10	28	15	21	26	38	34	15	17	15	25	16	36
	35	12	31	8	18	7	24	29	49	18	24	13	13	15	24	38	28	41	16	26	8	16	16	34
	18	6	20	5	19	10	14	22	32	11	29	17	33	21	29	38	40	59	13	16	14	27	9	19
	23	7	10	6	21	10	6	13	28	19	18	6	24	24	22	31	21	37	22	27	21	22	5	19
	32	16	49	22	12	14	21	25	32	15	31	19	64	49	19	34	15	41	29	35	26	38	1	18
Percent of cells	70.9%	29.1%	70.4%	29.6%	72.1%	27.9%	38.1%	61.9%	66.9%	33.1%	64.8%	35.2%	62.1%	37.9%	42.7%	57.3%	41.0%	59.0%	42.0%	58.0%	37.2%	62.8%	38.9%	61.1%

Table S1. Primary rat Kupffer cell death following exposure to stimuli. Primary rat Kupffer cells were exposed to no stimuli (naïve), uninfected salivary gland extracts (sg), *P. berghei* mCherry sporozoites (Pb), or 387.5 ng/ml listeriolysin O, a pore forming toxin (LLO). After 15 minutes, the live/dead imaging reagent mix was added to the cells. Cells were imaged and the number of live and dead cells counted at 30 minutes, 1.5 hours, and 3 hours following cell exposure to stimuli. For each stimulus, three biological replicates (indicated by Coverslips 1, 2, and 3) were analyzed and five microscope fields using the 10× objective on the EVOS Cell Imaging System of each replicate was counted at each time point.
